# Deciphering the Molecular Mechanism Underlying African Animal Trypanosomiasis by Means of the 1000 Bull Genomes Project Genomic Dataset

**DOI:** 10.3390/biology11050742

**Published:** 2022-05-13

**Authors:** Abirami Rajavel, Selina Klees, Yuehan Hui, Armin Otto Schmitt, Mehmet Gültas

**Affiliations:** 1 Breeding Informatics Group, Department of Animal Sciences, Georg-August University, Margarethe von Wrangell-Weg 7, 37075 Göttingen, Germany; selina.klees@uni-goettingen.de (S.K.); yuehan.hui@stud.uni-goettingen.de (Y.H.); armin.schmitt@uni-goettingen.de (A.O.S.); 2 Center for Integrated Breeding Research (CiBreed), Georg-August University, Carl-Sprengel-Weg 1, 37075 Göttingen, Germany; 3 Faculty of Agriculture, South Westphalia University of Applied Sciences, Lübecker Ring 2, 59494 Soest, Germany

**Keywords:** African trypanosomiasis, Boran, N’Dama, regulatory SNPs, gene expression profiles, downstream effectors

## Abstract

**Simple Summary:**

Climate change is increasing the risk of spreading vector-borne diseases such as African Animal Trypanosomiasis (AAT), which is causing major economic losses, especially in sub-Saharan African countries. Mainly considering this disease, we have investigated transcriptomic and genomic data from two cattle breeds, namely Boran and N‘Dama, where the former is known for its susceptibility and the latter one for its tolerance to the AAT. Despite the rich literature on this disease, there is still a need to investigate underlying genetic mechanisms to decipher the complex interplay of regulatory SNPs (rSNPs), their corresponding gene expression profiles and the downstream effectors associated with the AAT disease. The findings of this study complement our previous results, which mainly involve the upstream events, including transcription factors (TFs) and their co-operations as well as master regulators. Moreover, our investigation of significant rSNPs and effectors found in the liver, spleen and lymph node tissues of both cattle breeds could enhance the understanding of distinct mechanisms leading to either resistance or susceptibility of cattle breeds.

**Abstract:**

African Animal Trypanosomiasis (AAT) is a neglected tropical disease and spreads by the vector tsetse fly, which carries the infectious *Trypanosoma* sp. in their saliva. Particularly, this parasitic disease affects the health of livestock, thereby imposing economic constraints on farmers, costing billions of dollars every year, especially in sub-Saharan African countries. Mainly considering the AAT disease as a multistage progression process, we previously performed upstream analysis to identify transcription factors (TFs), their co-operations, over-represented pathways and master regulators. However, downstream analysis, including effectors, corresponding gene expression profiles and their association with the regulatory SNPs (rSNPs), has not yet been established. Therefore, in this study, we aim to investigate the complex interplay of rSNPs, corresponding gene expression and downstream effectors with regard to the AAT disease progression based on two cattle breeds: trypanosusceptible Boran and trypanotolerant N’Dama. Our findings provide mechanistic insights into the effectors involved in the regulation of several signal transduction pathways, thereby differentiating the molecular mechanism with regard to the immune responses of the cattle breeds. The effectors and their associated genes (especially *MAPKAPK5*, *CSK*, *DOK2*, *RAC1* and *DNMT1*) could be promising drug candidates as they orchestrate various downstream regulatory cascades in both cattle breeds.

## 1. Introduction

Trypanosomiasis is a deadly neglected tropical disease that affects the health of several mammalian species, including cattle, horses and humans. When it affects the health of humans, this disease is commonly known as ‘sleeping sickness’ [1]. On the other hand, African Animal Trypanosomiasis (AAT), also known as nagana (which means ’useless’ in the Zulu language), affects the health of livestock and it is spread by the tsetse fly carrying salivarian trypanosomes [2,3,4]. It prevails extensively in 40 sub-Saharan African countries and accounts for huge economic losses to farmers, particularly affecting meat and milk production [5,6]. Therefore, it has gained socio-economic importance as it retards the agricultural development of several regions in those areas [7]. Particularly, AAT is caused by different *Trypanosoma* species, including *Trypanosoma congolense*, *Trypanosoma vivax* and *Trypanosoma brucei* spp. [7]. Out of them, *Trypanosoma congolense* is regarded as the most serious pathogen for livestock. In humans, these unicellular protozoans cause various diseases; for example, *T. brucei* causes sleeping sickness, which alters the sleep-wake cycle by interfering the circadian rhythm [8,9], whereas *T. cruzi* causes Chagas disease or American trypanosomiasis [10,11].

Trypanosomes infect a wide range of hosts and are transmitted into the bloodstream of the mammalian host [12,13,14,15]. When the tsetse fly transmits the trypanosomes into the body of the cattle, the parasite first infects the skin resulting in the lesions due to local host immune responses. Afterwards, it enters the blood circulation via lymphatic vessels [16,17,18,19]. Important symptoms primarily observed in animals after being infected with the most pathogenic *T. congolense* include anaemia, loss of body conditions, thrombocytopenia [20], lymphopenia, immunosuppression [21,22,23] and other secondary infections [24].

Few West African cattle breeds like N’Dama can control the development of the disease AAT, in contrast to the other breeds such as Boran [25]. As a trait, trypanotolerance is the ability to control parasitemia (development of parasites) and the associated anaemia [12,13,14,15]. Harnessing the genetic potential of trypanotolerant breeds like N’Dama, recent studies [26,27,28,29] have focussed on investigating the trait of trypanotolerance.

Mainly considering the trait of trypanotolerance, several researchers [29,30,31,32,33,34,35] have performed different types of analysis based on either gene expression data sets or genotype × phenotype data sets from the cattle breeds, namely trypanosusceptible Boran and trypanotolerant N’Dama (for a short overview, see [26,36]). Among these previous studies [29,30,31,32,33,34,35], especially, Hanotte et al. [30] performed genome-wide analyses and identified genomic regions to reveal the genetic differences between the cattle breeds related to the trait of trypanotolerance. In this regard, Noyes et al. [34] analysed the gene expression dataset to identify differentially expressed genes that responded to trypanosome infection to differentiate between the susceptible and tolerant cattle breeds. To this end, Mekonnen et al. [29] investigated the genetic background of N’Dama along with other cattle breeds. Moreover, O’Gorman et al. [33] and Gautier et al. [35] conducted the genetic and expression analyses to identify the significant chromosomal regions which could affect the susceptibility/tolerance of the cattle breeds.

To decipher the underlying regulatory mechanisms determining trypanosusceptibility/trypanotolerance of these cattle breeds, we have recently analysed a time-series gene expression data set of the two cattle breeds [37,38]. Particularly, by considering the AAT disease development as a multi-stage progression process, we investigated Monotonically Expressed Genes (MEGs) to capture the complete progression process of the disease. As a result of our previous studies [37,38], we reported several transcription factors (TFs), their co-operations and master regulators governing the upstream molecular mechanism during the infection. Despite the rich literature on this disease, there is still a need for further investigation of genetic mechanisms of the regulatory processes addressing the complex interplay between regulatory SNPs, their corresponding gene expression and the downstream effectors in association with the AAT disease.

Recent progress in molecular biology created the opportunity to use heterologous animal models to investigate complex traits and genetics underlying the disease mechanisms [39,40,41]. Remarkably, integratomics is fast becoming the latest trend in omics research while integrating a variety of omics data (such as genomic, transcriptomic and proteomic data), irrespective of the species [42]. Access to genome sequences of species like cattle unlocked the potential for integrating transcriptomic and genomic data. The information about effectors, which are end products located several steps downstream and regulate the functioning of multiple signal transduction pathways, is pivotal for understanding the complex molecular mechanisms such as the response of the cell to an extracellular pathogen. In silico study of the candidate, MEGs were undertaken to identify the novel trypanotolerance-associated rSNPs and downstream effectors. The candidate MEGs from our analysis of effectors were analysed for their gene expression profiles.

To address this missing point of previous studies, we applied an integratomics approach to study the complex interplay of biological processes orchestrated by rSNPs, genes and downstream effectors during the AAT disease progression. For this purpose, we performed integrated systems biology and bioinformatics approaches while incorporating the transcriptomic data [34] and genomic data from the 1000 Bull Genome Project [43] for both cattle breeds. To examine the combinatorial interplay, we firstly identified the regulatory SNPs (rSNPs), which are located in the promoter regions of the MEGs and which, as per definition, exert a strong influence on the binding affinity of the TFs either by the deletion or the creation (gain/loss) of a transcription factor binding site (TFBS) [44,45,46]. In accordance with previous studies on the rSNPs [47,48], it is today well-known that the rSNPs based on their consequences can influence and change individual steps of gene expression. Subsequently, we extracted for each tissue (liver, spleen and lymph node) the MEGs harbouring the regulatory SNPs in their promoters by manually studying their gene expression profiles during the AAT disease progression. Finally, we explored the corresponding downstream effectors that have a pronounced effect on the activation and regulation of a multitude of downstream signalling pathways. Taken together, our findings provide a multifaceted glimpse of (i) the regulatory SNPs governing the susceptibility/tolerance mechanism of the cattle breeds; (ii) downstream effectors associated with the MEGs harbouring rSNPs, and their biological and immune-related functions, which could potentially distinguish the susceptibility/tolerance mechanism of cattle breeds to AAT disease; (iii) deciphering novel hypotheses and potential targets for breeding goals and therapeutic implications.

## 2. Materials and Methods

**Figure 1:**
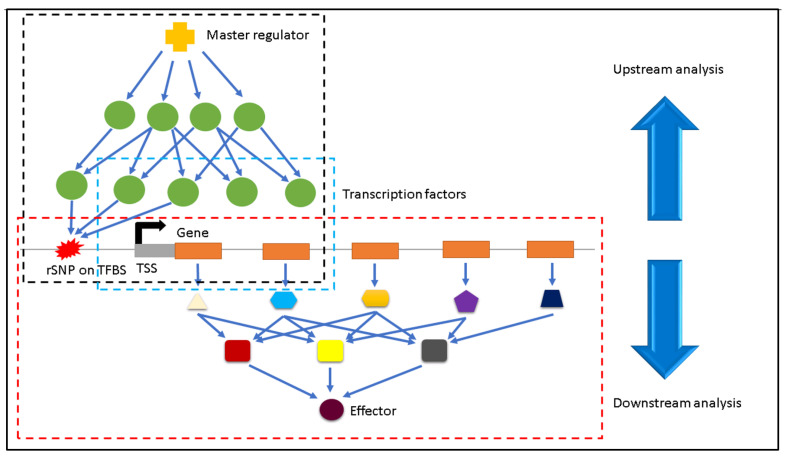
Overview of analyses. Our first computational study (middle box in blue-dotted lines) highlighted the transcription factor co-operations associated with the AAT disease progression [37]. In our second study (top box in black dashed lines), we performed an upstream analysis to detect master regulators and over-represented upstream pathways related to AAT [38]. In the current study (bottom box in red dashed lines), we focus on the downstream analysis to decipher the complex interplay of regulatory SNPs (rSNPs), their related gene expression and their corresponding downstream effectors, which regulate a multitude of signal transduction pathways during the AAT disease progression.

In this section, we illustrate an overview of the analyses to highlight the difference between our previous studies [37,38] and the current study. Simultaneously this overview shows how this present study complements our previous studies. Figure 1 outlines the overview of our analyses.

### 2.1. Monotonically Expressed Genes

In this study, we investigate the complex interplay of regulatory SNPs (rSNPs), the related gene expression and their corresponding downstream effectors.

A time-series microarray data set, originally published by Noyes et al. (http://www.ebi.ac.uk/arrayexpress/, accession no. E-MEXP-1778, accessed on 12 March 2019) [34], has been analysed [37] to identify the Monotonically Expressed Genes (MEGs), expressed either with increasing or decreasing patterns during a biological process or a disease. The data set consisted of the gene expression recordings from three tissues (liver, spleen and lymph node) of two cattle breeds: trypanotolerant N’Dama and trypanosuceptible Boran. In this experiment, tissue harvest was performed on days 0, 21 and 35. Only the liver tissue samples were collected at additional time points such as days 12, 15, 18, 26, 29 and 32. Readers who are interested in this analysis and the identification of MEGs are kindly referred to [37].

We use these identified MEGs in our further analysis. The numbers of MEGs are provided in Table 1 and the lists of MEGs are provided in Supplementary File S1.

### 2.2. Genotype Data

The genotype-phenotype data set of the cattle breeds Boran and N’Dama used in this study are a part of the 1000 Bull Genomes Project [43].

The genotype data contains for 23 animals (11 Boran and 12 N’Dama) 783,637 variants that are located in the promoter regions covering from −1000 bp to 0 bp relative to the transcription start sites of the MEGs. Furthermore, we considered the resistance of the cattle breed as a qualitative phenotype and assigned ‘0’ and ‘1’ to represent the disease phenotypes for resistance and susceptibility, respectively. Similar to our previous studies [46,49], for the purpose of quality control, filtering of genotype data was then carried out to remove the SNPs with a minor allele frequency (MAF) less than 0.1, call rate less than 0.95 and which significantly deviated from Hardy–Weinberg Equilibrium (*p* < 1 × 10^−8^). After this filtering, the data set contained about 19,330 SNPs and 23 animals for further analyses. We performed a Genome-Wide Association analysis using PLINK 1.9 software [50]. The genotype-phenotype association was evaluated with PLINK by chi-squared allelic test. As suggested by Heinrich et al. [46], we used the false discovery rate (FDR) of 0.1 to control the type I error rate.

### 2.3. Identification of Regulatory SNPs

In previous studies [44,46], an SNP is defined to be a regulatory SNP (rSNP) if it is located in the promoter region of a gene and if it affects the binding affinity of one or more transcription factors (TFs) to their respective binding sites which leads to the gain/loss of TFBSs. According to the rSNP detection pipeline, we extracted the flanking sequence of ±25 bp for each selected SNP. Finally, we scanned the flanking sequences of the SNPs for both alternate and reference alleles using the MATCH${}^{\mathrm{TM}}$ program [51] and thus classified an SNP as rSNP if it leads to gain and loss of a TFBS.

### 2.4. Finding the Effectors

Taking the rSNPs into account, we filtered the list of MEGs under study that harbour at least one rSNP within their promoter. Using the filtered list of MEGs for each tissue individually, we employed the systems biology platform geneXplain [52] to identify the effector molecules. Effectors are important signalling molecules that are end products located several steps downstream and regulate the functioning of a multitude of signalling cascades. With regard to AAT disease, the knowledge about the effectors could provide promising information to decipher their complex interplay with rSNPs and the corresponding MEGs. The identification of effectors was performed using the ‘Effector search’ function on the geneXplain platform, which utilises the TRANSPATH^®^ database [53] for searching the downstream effectors regulated by the input set of MEGs.

## 3. Results and Discussion

By analysing regulatory SNPs (rSNPs), the related gene expression profiles of MEGs and their associated downstream effectors, we established their complex interplay involved in the AAT disease progression for both cattle breeds. For this purpose, we firstly performed a genome-wide association analysis and obtained 19,330 significant SNPs, out of which 1849 SNPs have been further classified as rSNPs.

Uncovering disease-related SNPs is recently gaining utmost importance as they can have an impact on the disease progression and also on how the infected individual responds to the infection [54,55,56,57,58]. In particular, rSNPs are of great interest as they could be causal and thus alter the protein-DNA interaction. Afterwards, considering the MEGs of interest, which harbour at least one rSNP in their promoter regions, we created for each tissue a filtered list of monotonically expressed genes. Finally, using these lists of MEGs obtained for each tissue (liver, spleen and lymph node) for both cattle breeds, we identified the downstream effectors to investigate further the underlying molecular mechanisms that orchestrate differences in the level of tolerance of the cattle breeds to AAT. The numbers of rSNPs and MEGs of interest are given in Table 2 and Table 3, respectively. The list of respective rSNPs and MEGs are provided as Supplementary Files S2 and S3.

### 3.1. Identification of Downstream Effectors

We employed the “Effector Search” algorithm from the geneXplain platform [52] using the tissue-based MEG sets of interest for the computational identification of downstream effectors. From this analysis, we obtained a total of 18 effectors that are unique for the breeds and the three tissues (given in Table 4). Remarkably, the effectors obtained are completely different between the susceptible and tolerant cattle breeds.

### 3.2. Downstream Effectors for Liver Tissue

The analysis of the MEGs for the liver tissue resulted in the detection of three effectors for Boran (namely SRF, PKCδ and a complex of proteins ITK, LCK, PLCγ and SLP76) and N’Dama (p85α, chTOG:H3F3A and TF2-1).

Serum response factor (c-fos serum response element-binding transcription factor) is a transcription factor belonging to the MADS (MCM1, Agamous, Deficiens and SRF) box superfamily. It is mainly involved in the regulation of immediate-early genes and takes part in important cellular processes like cell differentiation, cell growth and apoptosis. The gene encoding this protein serves as the major target for several signalling pathways, in particular, the mitogen-activated protein kinase pathway (MAPK) that plays a significant role in the immune surveillance mechanism supporting the trypanosome infection [59]. Therefore, the SRF protein could be directing the immune evasion, thereby assisting susceptibility of the cattle breed in AAT disease progression.

The second effector, PKCδ, found in Boran’s liver tissue, has been reported as the marker of inflammation and plays an essential role in tuberculosis disease progression in humans [60]. This could be an important hint for the AAT disease progression in the susceptible cattle breed Boran. Moreover, the third effector consists of four proteins, namely ITK, SLP 76, LCK and PLCγ1. Inducible T-cell kinase (ITK) belongs to the Tec family of non-receptor tyrosine kinases, which are expressed in immune cells like mast cells and T cells. It plays a critical role in T-lymphocyte development and functioning and is involved in regulating T-cell receptor signalling. Furthermore, it is activated with respect to antigen receptors, for example, T-cell receptor stimulation [61,62,63]. It is reported to be important for the replication of the virus inside the infected host cells [64], elucidating its role in supporting the pathogen infection in AAT. SH2-domain-containing leukocyte protein of 76 kDa (SLP 76) is one of the key adaptor proteins expressed only in the haematopoietic part of the immune cells such as monocyte, granulocyte and T lymphocyte lineage [65]. The protein SLP 76 plays a crucial role in the regulation of several signalling cascades [66]. Additionally, its expression is regulated during T cell maturation and activation [65]. This demonstrates the close association of the protein SLP 76 with the haematopoiesis and generation of immune responses relating to anaemia in AAT disease, an important hallmark of AAT. The association of LCK (lymphocyte-specific cytoplasmic protein-tyrosine kinase) to CD4 and CD8 is necessary for antigen-specific T cell development and activation [67]. Of particular interest, phospholipase C gamma 1 (PLCγ1) signalling is important for several physiological processes like cell differentiation [68,69].

In our analysis, we found an effector as a complex of chTOG and H3F3A for the liver tissue of N’Dama. The chTOG is a human TOG protein, reported as a mitotic error correction factor playing an important role in accurate chromosome segregation during cell division [70]. Further, H3F3A belongs to the group of basic nuclear histone proteins supporting the structure of the chromosome, thereby maintaining the genome integrity [71]. Another effector, TF2-1, found in the liver tissue of N’Dama, is a non-infectious and intracellular retrotransposon [72]. However, both of these effectors were not illustrated in relation to host-pathogen interaction, and thus, their potential roles in AAT disease progression are not studied. On the other hand, the third effector p85 α, is an adapter subunit of heterodimer phosphatidylinositol 3-kinase, which is involved in the production of phospholipids. By interacting with other proteins such as p110 α and PTEN, p85 α could regulate the PI3K pathway either in a positive or negative manner [73]. Due to the importance of the phosphatidylinositol 3-kinase (PI3K) signalling pathway in many diseases [74], the regulatory activity of p85 α is gaining importance in response to infections as well. This demonstrates the role of p85 α during AAT infection, which might play a crucial part in trypanotolerance of N’Dama by maintaining the lipid synthesis in the host’s liver intact without interruption from the pathogenic attack.

### 3.3. Downstream Effectors for Spleen

The analysis of the effectors for spleen tissue unravelled p53:HEXIM1, HEXIM1:p53 and histone H3:DNA-PKcs for Boran and LYZL2 isoform 2:LRP11, PON-2 isoform 1:LRP11 and WSX-1:LRP11 for N’Dama.

The first two effectors are a complex of two proteins: HEXIM1 and p53. Hexamethylene bisacetamide-inducible protein 1 (HEXIM1) protein encoded by *HEXIM1* is known for its role in the regulation of gene expression, especially with regard to innate immunity [75]. Particularly, it has been reported in the *Trypanosoma cruzi* infection, in association with splenomegaly in the Hexim1^+/−^ mice. It was shown how the downregulation of *HEXIM1* protects the host against *T. cruzi* infection [76]. This hints at the functioning of HEXIM1 during the infection process by increasing inflammation. Another part of the protein complex, p53, identified for the spleen tissue, acts as a tumour suppressor protein in humans, therefore called as “guardian of the genome” [77,78]. In recent studies, it has been demonstrated that p53 regulates inflammation [79] which is highly associated with AAT. Especially in a study involving bacterial infection [80], deletion or inhibition of p53 resulted in the clearance of extracellular bacteria, which reveals the regulatory role of p53 in the defence against extracellular pathogens establishing the modulation of microbicidal function. Another effector found in the spleen tissue, DNA Protein Kinase, has been reported for its roles in regulating metabolic pathways, particularly in fatty acid synthesis [81]. It is one of the key players responding to DNA damage and in IRF-3-dependent innate immunity [82]. Especially, DNA Damage Response PK has been studied as a driver in evading host immunity [83] and in developmental transitions occurring between the vector and the host [84]. This effector could play a role in immune evasion, thereby supporting the trypanosome infection and increasing the susceptibility of Boran.

For the spleen tissue of N’Dama, the identified effectors, including LYZL2 isoform 2, PON2 isoform 1 and WSX1 are complexes of LRP11 protein. LRP11 plays a key role in the development of stress responses in mice, as suggested by Xu et al. in [85]. It is well-known that through the activation of the stress response, the host’s body provides energy immediately available for immune responses against the parasitic infection, therefore benefitting the host to recover earlier [86]. LYZL2 identified as one of the effectors, exhibits lysozyme activity, which functions as bacteriolytic factors [87] and they are mainly involved in the host defence. Their biological function in relation to parasitic infection has not been largely studied yet. Interestingly, we found Paraoxonase 2 (PON2) in the spleen tissue of N’Dama, which is an intracellular membrane protein exerting anti-oxidant functions [88]. Macrophages are key players against extracellular and intracellular pathogens. In this regard, PON2 has been studied for their expression in the macrophages [89]. In a study involving bacterial infection with *Pseudomonas aeroginosa*, the role of PON2 in the innate immune defence has been demonstrated [90]. The next effector, WSX1, is a class I cytokine receptor for IL27 and is predominantly expressed in lymphoid tissues like the spleen and lymph nodes [91]. Being the IL27 receptor, WSX1 has been studied to be associated with the IL27 signalling pathway. It is further involved in the regulation of Th1-type adaptive immune responses and also of the cells of the innate immune system [92]. Villarino et al. reported in their study [93] that WSX1 is necessary for resistance to parasitic infection from *Toxoplasma gondii*. Particularly, this could provide an important hint on the functioning of WSX1 in resistance of N’Dama to AAT disease.

### 3.4. Downstream Effectors for Lymph Node

The analysis of the MEGs of lymph node tissue reveals the effectors, namely LIMP-2:Prpf8, VICKZ3:Prpf8 and SNRPGP15:Prpf8, for Boran and the effectors Ssu72, MTMR4, Clathrin LCb for N’Dama.

Considering the biological roles of effector LIMP-2, it is a type III glycoprotein belonging to the CD36 superfamily of scavenger proteins, facilitating the transport of the acid hydrolase β-glucocerebrosidase (GC) [94]. This protein provides a strong connection between cholesterol export and innate immunity [95,96] as lipids play crucial roles in the multiplication of the trypanosome infection cycle. Therefore, the LIMP2 protein might be a strong candidate protein crucial for establishing the infection, thereby making the cattle breed Boran susceptible to AAT. Another effector, VICKZ3, for the lymph node issue of Boran, belongs to the family of RNA binding proteins and is expressed in the developing central nervous system [97] during embryogenesis. This group of proteins are associated with the regulation of RNA and are involved in controlling the cellular processes like proliferation and translational repression [98]. Furthermore, the effector SNRPGP15 (Small Nuclear Ribonucleoprotein G-like protein 15) is a part of the spliceosome, which mainly takes part in RNA metabolism [99]. Finally, part of the protein complexes of all the three effectors is pre-mRNA processing factor 8 (Prpf8) is a highly conserved protein and known for its role in the pre-mRNA splicing process [100]. However, VICKZ3, SNRPGP15 and PRPF8 have not been largely studied in terms of host-pathogen interaction; therefore, their potential role in AAT disease progression is currently unknown.

On the other hand, the effectors identified for the lymph node tissue of N’Dama suggest their crucial roles in immunity, bolstering the host’s defence against the parasite. The effector Ssu72 is a dual protein phosphatase that plays a role in RNA processing. A recent study has associated the Ssu72 protein in macrophages with the process of immunometabolism [101], implicating a closer connection between immunity and trypanotolerance of N’Dama. The next effector, Myotubularin-related protein 4 (MTMR4), is an intracellular protein that exhibits lipid and protein phosphatase activities in several cellular functions. Especially MTMR4 is involved in the negative regulation of TGF-β signalling. During the infection of *Trypanosoma cruzi*, the role of TGF-β has been demonstrated to inhibit the functioning of immune effector cells and the production of interferon α, thereby resulting in the multiplication of the pathogen [102]. Therefore, MTMR4 indirectly assists the host in decreasing the pathogen numbers within the body, supporting the tolerance mechanism of the cattle breed N’Dama. Another effector, Clathrin, is a cytosolic protein made up of heavy and light chains. Clathrin light chains (LCb) are important components of clathrin-coated vesicles, especially necessary to uptake large foreign particles into the vesicles [103]. This effector found in lymph nodes could represent the engulfing of infectious parasites during the AAT disease in the body of N’Dama.

In particular, the knowledge of these effectors provides essential information in distinguishing the downstream events underlying the susceptibility and tolerance mechanisms of the cattle breeds Boran and N’Dama, respectively. Further validation of the results from the molecular biology end is necessary to evaluate the biological importance of their functions in the AAT disease progression as well as to gain a comprehensive understanding of their roles in susceptibility/tolerance mechanisms of the cattle breeds.

### 3.5. Gene Expression Profile Analysis of MEGs Harbouring rSNPs

Using gene expression profiles, it is possible to gain insights into the differences in the expression levels under certain cellular conditions. Therefore, we were additionally interested in the gene expression profiles for the MEGs of interest to decipher their differentiation between the cattle breeds. For this purpose, we manually analysed and then annotated the gene expression profiles of MEGs for each tissue to investigate their expression patterns. A closer look at these gene expression profiles reveals the distinguishing expression patterns for five MEGs (namely *MAPKAPK5*, *CSK*, *DOK2*, *RAC1* and *DNTM1*) expressed over several time points in the liver tissue of both breeds Boran and N’Dama (see Supplementary File S4). Interestingly, these genes are key players in the detection of effectors found in liver tissue (see Supplementary File S4). Gene expression profiles of other MEGs of interest are provided in Supplementary File S5.

**Figure 2:**
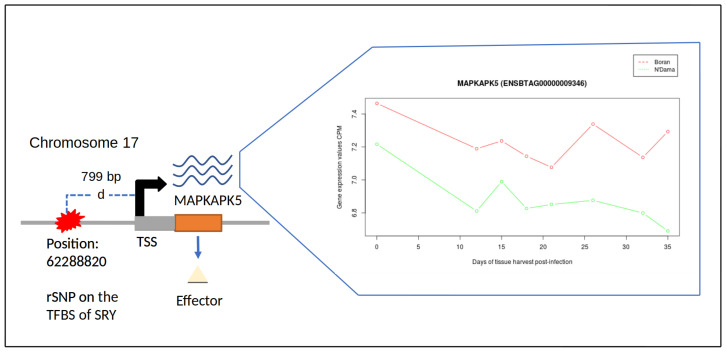
An overview of gene expression profile analysis. Schematic representation of rSNP at position 62,288,820 bp of chromosome 17 influencing the gene expression pattern of *MAPKAPK5*. ‘d’ refers to the distance of the rSNP from the transcription start site (TSS).

Figure 2 exemplarily shows the changes in the gene expression profile of *MAPKAPK5* for liver tissue of both cattle breeds, harbouring rSNPs in its promoter region. Considering the biological roles, MAPKAPK5 (MAPK Activated Protein Kinase 5), encoded by the gene *MAPKAPK5*, is a serine/threonine-protein kinase that plays a major role in the post-transcriptional regulation of MYC, [104,105] which is intimately associated with immune evasion [106]. The protein encoded by the gene *CSK* plays a critical role in the activation of T-cells and is involved in several pathways, which include the regulation of Src family kinases [107]. Expression of *DOK2* has been reported to regulate the cell cycle of haematopoietic stem cells. Furthermore, the inactivation of *DOK2* also resulted in the aberrant activation of MAP kinase [108], implicating that their functional loss could exacerbate the AAT disease. The protein encoded by *RAC1* (Rac Family Small GTPase 1) is important in regulating cellular processes like phagocytosis of apoptotic cells and binds to effector proteins in their active state [109]. *DNMT1* plays a critical part in regulating the immune system and is regarded indispensable for the inhibition of Foxp3+Treg cells [110].

## 4. Conclusions

Transcription factors are involved in regulating transcription processes by binding to short DNA sequences called transcription factor binding sites (TFBSs). In particular, single nucleotide polymorphisms (SNPs) are widely studied with regard to the disease mechanisms as they can have direct control over the disease susceptibility (causal polymorphisms). Importantly, regulatory SNPs (rSNPs) that are located in the regulatory regions like promoters can significantly affect the gene expression, especially by modifying the binding sites of the TFs. Knowledge about the rSNPs and their complex interplay with the corresponding gene expression and downstream effectors could reveal multiple disease-associated polymorphisms, which can be further used as targets in drug design and breeding programs. Taking the importance of rSNPs and their combinatorial interplay into account, we performed a systematic investigation of genomic and transcriptomic data of two cattle breeds, Boran and N’Dama, to unravel the underlying genetic mechanisms of AAT disease progression. Our findings provide mechanistic insights into significant rSNPs, which are harboured within the promoter regions of MEGs. Moreover, our further investigation of effectors found in the liver, spleen and lymph node tissues of both cattle breeds enhanced our understanding of distinct mechanisms leading to either resistance or susceptibility of cattle breeds. Our current study complements our previous studies, which mainly focused on the upstream events, including TFs and their co-operations as well as master regulators. Taken together, our findings provide a multifaceted glimpse of (i) novel insights into the rSNPs governing the susceptibility/tolerance mechanism of the cattle breeds; (ii) downstream effectors, particularly LYZL2, WSX1 and MTMR4 and their biological roles related to innate and adaptive immune responses during the AAT disease progression.

## Figures and Tables

**Table 1:** Numbers of statistically significant Monotonically Expressed Genes in ascending and descending order for liver-, spleen- and lymph node-tissues for the cattle breeds Boran and N’Dama.

	Boran		N’Dama	
	**Ascending**	**Descending**	**Ascending**	**Descending**
Liver	741	308	757	124
Spleen	669	126	13	139
Lymph node	87	5	119	114

**Table 2:** Numbers of regulatory SNPs found for liver-, spleen- and lymph node-tissues for the cattle breeds Boran and N’Dama.

	Boran		N’Dama	
	**Gain of TFBS**	**Loss of TFBS**	**Gain of TFBS**	**Loss of TFBS**
Liver	365	403	342	385
Spleen	152	154	3	8
Lymph node	10	12	3	12

**Table 3:** Numbers of MEGs under study harboring at least one rSNP in their promoter region, for liver-, spleen- and lymph node-tissues for the cattle breeds Boran and N’Dama.

	Boran	N’Dama
Liver	194	102
Spleen	157	9
Lymph node	13	5

**Table 4:** Downstream effectors obtained for liver-, spleen- and lymph node-tissues for the cattle breeds Boran and N’Dama.

Cattle Breed	Tissue	Effectors
Boran	Liver	Itk:Lck:PLCgamma1:SLP-76
Boran	Liver	PKCdelta
Boran	Liver	SRF
Boran	Spleen	histone H3:DNA-PKcs
Boran	Spleen	p53:HEXIM1
Boran	Spleen	HEXIM1:p53
Boran	Lymph node	LIMPII:Prpf8
Boran	Lymph node	VICKZ3:Prpf8
Boran	Lymph node	SNRPGP15:Prpf8
N’Dama	Liver	CHTOG:h3f3a
N’Dama	Liver	p85alpha
N’Dama	Liver	TFII-I
N’Dama	Spleen	LYZL2-isoform2:LRP11
N’Dama	Spleen	PON 2-isoform1:LRP11
N’Dama	Spleen	WSX-1:LRP11
N’Dama	Lymph node	Ssu72
N’Dama	Lymph node	MTMR4
N’Dama	Lymph node	Clathrin LCb

## Data Availability

Not applicable.

## Supplementary Materials

The following supporting information can be downloaded at https://www.mdpi.com/article/10.3390/biology11050742/s1, Supplementary File S1: Lists of Monotonically Expressed Genes obtained from the MFSelector approach. Supplementary File S2: Lists of regulatory SNPs obtained for liver-, spleen- and lymph node tissues of Boran and N’Dama. Supplementary File S3: List of breed-specific and tissue-specific MEGs harbouring regulatory SNPs in their promoter regions. Supplementary File S4: Gene expression profiles of monotonically expressed genes *MAPKAPK5*, *CSK*, *DOK2*, *RAC1* and *DNTM1* that harbour regulatory SNPs in their promoter regions. Supplementary File S5: Lists of QR codes of videos for gene expression profiles corresponding to the MEGs harbouring rSNPS associated with liver-, spleen- and lymph node tissues of Boran and N’Dama.

## References

[1] Brun R., Blum J. (2012). Human African trypanosomiasis. Infect. Dis. Clin. N. Am..

[2] Barrett M.P., Boykin D.W., Brun R., Tidwell R.R. (2007). Human African trypanosomiasis: Pharmacological re-engagement with a neglected disease. Br. J. Pharmacol..

[3] Kappmeier K., Nevill E., Bagnall R. (1998). Review of Tsetse Flies and Trypanosomosis in South Africa. Onderstepoort J. Vet. Res..

[4] Van den Bossche P. (2001). Some general aspects of the distribution and epidemiology of bovine trypanosomosis in southern Africa. Int. J. Parasitol..

[5] Firesbhat A., Desalegn C. (2015). Epidemiology and impacts of trypanosomosis in cattle. Eur. J. Appl. Sci..

[6] Radostits O.M., Gay C.C., Hinchcliff K.W., Constable P.D. (2006). Veterinary Medicine E-Book: A Textbook of the Diseases of Cattle, Horses, Sheep, Pigs and Goats.

[7] Losos G.J., Ikede B. (1972). Review of pathology of diseases in domestic and laboratory animals caused by *Trypanosoma congolense*, *T. vivax*, *T. brucei*, *T. rhodesiense* and *T. gambiense*. Vet. Pathol..

[8] Buguet A., Bert J., Tapie P., Tabaraud F., Doua F., Lonsdorfer J., Bogui P., Dumas M. (1993). Sleep-wake cycle in human African trypanosomiasis. J. Clin. Neurophysiol..

[9] Rijo-Ferreira F., Carvalho T., Afonso C., Sanches-Vaz M., Costa R.M., Figueiredo L.M., Takahashi J.S. (2018). Sleeping sickness is a circadian disorder. Nat. Commun..

[10] Rassi A., Rassi A., Marin-Neto J.A. (2010). Chagas disease. Lancet.

[11] Rassi A., de Rezende J.M. (2012). American trypanosomiasis (Chagas disease). Infect. Dis. Clin..

[12] D’Ieteren G., Authié E., Wissocq N., Murray M. (1998). Trypanotolerance, an option for sustainable livestock production in areas at risk from trypanosomosis. Rev. Sci. Tech..

[13] Roelants G. (1986). Natural resistance to African trypanosomiasis. Parasite Immunol..

[14] Authie É. (1993). Contribution à L’étude des Mécanismes Immunologiques Impliqués dans la Trypanotolérance des Taurins d’Afrique. Ph.D. Thesis.

[15] Ellis J., Scott J., MacHugh N.D., Gettinby G., Davis W. (1987). Peripheral blood leucocytes subpopulation dynamics during *Trypanosoma congolense* infection in Boran and N’Dama cattle: An analysis using monoclonal antibodies and flow cytometry. Parasite Immunol..

[16] Mulligan H.W., Porrs W. (1970). The African Trypanosomiases.

[17] Luckins A., Gray A. (1978). An extravascular site of development of *Trypanosoma congolense*. Nature.

[18] Emery D., Moloo S. (1981). The dynamics of the cellular reactions elicited in the skin of goats by Glossina morsitans morsitans infected with Trypanosoma (Nannomonas) congolense or T. (Duttonella) vivax. Acta Trop..

[19] Akol G., Murray M., Hirumi H., Hirumi K., Moloo S. (1986). Infectivity to cattle of metacyclic forms of Trypanosoma (Nannomonas) congolense propagated in vitro. I. Development of localized skin reactions following intradermal inoculation. Acta Trop..

[20] Wellde B.T., Kovatch R.M., Chumo D.A., Wykoff D.E. (1978). *Trypanosoma congolense*: Thrombocytopenia in experimentally infected cattle. Exp. Parasitol..

[21] Rurangirwa F., Tabel H., Losos G., Masiga W., Mwambu P. (1978). Immunosuppressive effect of *Trypanosoma congolense* and *Trypanosoma vivax* on the secondary immune response of cattle to *Mycoplasma mycoides* subsp *mycoides*. Res. Vet. Sci..

[22] Rurangirwa F., Tabel H., Losos G., Tizard I. (1979). Suppression of antibody response to Leptospira biflexa and Brucella abortus and recovery from immunosuppression after Berenil treatment. Infect. Immun..

[23] Rurangirwa F., Musoke A., Nantulya V., Tabel H. (1983). Immune depression in bovine trypanosomiasis: Effects of acute and chronic *Trypanosoma congolense* and chronic *Trypanosoma vivax* infections on antibody response to Brucella abortus vaccine. Parasite Immunol..

[24] Tabel H., Losos G., Maxie M. (1980). Experimental bovine trypanosomiasis (*Trypanosoma vivax* and *T. congolense*). II. Serum levels of total protein, albumin, hemolytic complement, and complement component C3. Tropenmedizin Und Parasitol..

[25] Starkey P.H. (1984). N’Dama cattle—A productive trypanotolerant breed. World Anim. Rev..

[26] Courtin D., Berthier D., Thevenon S., Dayo G.K., Garcia A., Bucheton B. (2008). Host genetics in African trypanosomiasis. Infect. Genet. Evol..

[27] Bahbahani H., Salim B., Almathen F., Al Enezi F., Mwacharo J.M., Hanotte O. (2018). Signatures of positive selection in African Butana and Kenana dairy zebu cattle. PLoS ONE.

[28] Tijjani A., Utsunomiya Y.T., Ezekwe A.G., Nashiru O., Hanotte O. (2019). Genome sequence analysis reveals selection signatures in endangered trypanotolerant West African Muturu cattle. Front. Genet..

[29] Mekonnen Y.A., Gültas M., Effa K., Hanotte O., Schmitt A.O. (2019). Identification of candidate signature genes and key regulators associated with Trypanotolerance in the Sheko Breed. Front. Genet..

[30] Hanotte O., Ronin Y., Agaba M., Nilsson P., Gelhaus A., Horstmann R., Sugimoto Y., Kemp S., Gibson J., Korol A. (2003). Mapping of quantitative trait loci controlling trypanotolerance in a cross of tolerant West African N’Dama and susceptible East African Boran cattle. Proc. Natl. Acad. Sci. USA.

[31] Hill E.W., O’Gorman G.M., Agaba M., Gibson J.P., Hanotte O., Kemp S.J., Naessens J., Coussens P.M., MacHugh D.E. (2005). Understanding bovine trypanosomiasis and trypanotolerance: The promise of functional genomics. Vet. Immunol. Immunopathol..

[32] Fisher P., Hedeler C., Wolstencroft K., Hulme H., Noyes H., Kemp S., Stevens R., Brass A. (2007). A systematic strategy for large-scale analysis of genotype–phenotype correlations: Identification of candidate genes involved in African trypanosomiasis. Nucleic Acids Res..

[33] O’Gorman G.M., Park S.D., Hill E.W., Meade K.G., Coussens P.M., Agaba M., Naessens J., Kemp S.J., MacHugh D.E. (2009). Transcriptional profiling of cattle infected with *Trypanosoma congolense* highlights gene expression signatures underlying trypanotolerance and trypanosusceptibility. BMC Genom..

[34] Noyes H., Brass A., Obara I., Anderson S., Archibald A.L., Bradley D.G., Fisher P., Freeman A., Gibson J., Gicheru M. (2011). Genetic and expression analysis of cattle identifies candidate genes in pathways responding to *Trypanosoma congolense* infection. Proc. Natl. Acad. Sci. USA.

[35] Gautier M., Flori L., Riebler A., Jaffrézic F., Laloé D., Gut I., Moazami-Goudarzi K., Foulley J.L. (2009). A whole genome Bayesian scan for adaptive genetic divergence in West African cattle. BMC Genom..

[36] Yaro M., Munyard K., Stear M., Groth D. (2016). Combatting African animal trypanosomiasis (AAT) in livestock: The potential role of trypanotolerance. Vet. Parasitol..

[37] Rajavel A., Heinrich F., Schmitt A.O., Gültas M. (2020). Identifying Cattle Breed-Specific Partner Choice of Transcription Factors during the African Trypanosomiasis Disease Progression Using Bioinformatics Analysis. Vaccines.

[38] Rajavel A., Schmitt A.O., Gültas M. (2021). Computational Identification of Master Regulators Influencing Trypanotolerance in Cattle. Int. J. Mol. Sci..

[39] Shook G. (2006). Major advances in determining appropriate selection goals. J. Dairy Sci..

[40] Ron M., Israeli G., Seroussi E., Weller J.I., Gregg J.P., Shani M., Medrano J.F. (2007). Combining mouse mammary gland gene expression and comparative mapping for the identification of candidate genes for QTL of milk production traits in cattle. BMC Genom..

[41] Ogorevc J., Kunej T., Razpet A., Dovc P. (2009). Database of cattle candidate genes and genetic markers for milk production and mastitis. Anim. Genet..

[42] Cannistraci C.V., Ogorevc J., Zorc M., Ravasi T., Dovc P., Kunej T. (2013). Pivotal role of the muscle-contraction pathway in cryptorchidism and evidence for genomic connections with cardiomyopathy pathways in RASopathies. BMC Med. Genom..

[43] Hayes B.J., MacLeod I.M., Daetwyler H.D., Bowman P.J., Chamberlian A., Vander Jagt C., Capitan A., Pausch H., Stothard P., Liao X. (2014). Genomic prediction from whole genome sequence in livestock: The 1000 bull genomes project. Proceedings of the World Congress of Genetics Applied to Livestock Production.

[44] Klees S., Lange T.M., Bertram H., Rajavel A., Schlüter J.S., Lu K., Schmitt A.O., Gültas M. (2021). In Silico Identification of the Complex Interplay between Regulatory SNPs, Transcription Factors, and Their Related Genes in *Brassica napus* L. Using Multi-Omics Data. Int. J. Mol. Sci..

[45] Klees S., Heinrich F., Schmitt A.O., Gültas M. (2021). agReg-SNPdb: A Database of Regulatory SNPs for Agricultural Animal Species. Biology.

[46] Heinrich F., Wutke M., Das P.P., Kamp M., Gültas M., Link W., Schmitt A.O. (2020). Identification of regulatory SNPs associated with vicine and convicine content of Vicia faba based on genotyping by sequencing data using deep learning. Genes.

[47] Buckland P.R. (2006). The importance and identification of regulatory polymorphisms and their mechanisms of action. Biochim. Biophys. Acta-Mol. Basis Dis..

[48] Jin H.J., Jung S., DebRoy A.R., Davuluri R.V. (2016). Identification and validation of regulatory SNPs that modulate transcription factor chromatin binding and gene expression in prostate cancer. Oncotarget.

[49] Ramzan F., Klees S., Schmitt A.O., Cavero D., Gültas M. (2020). Identification of Age-Specific and Common Key Regulatory Mechanisms Governing Eggshell Strength in Chicken Using Random Forests. Genes.

[50] Chang C.C., Chow C.C., Tellier L.C., Vattikuti S., Purcell S.M., Lee J.J. (2015). Second-generation PLINK: Rising to the challenge of larger and richer datasets. Gigascience.

[51] Wingender E., Chen X., Fricke E., Geffers R., Hehl R., Liebich I., Krull M., Matys V., Michael H., Ohnhaeuser R. (2001). Match-a tool for searching transcription factor binding sites in DNA sequences. Nucl. Acids Res..

[52] Stegmaier P., Kel A., Wingender E. (2017). geneXplainR: An R interface for the geneXplain platform. J. Open Source Softw..

[53] Krull M., Pistor S., Voss N., Kel A., Reuter I., Kronenberg D., Michael H., Schwarzer K., Potapov A., Choi C. (2006). TRANSPATH^®^: An information resource for storing and visualizing signaling pathways and their pathological aberrations. Nucleic Acids Res..

[54] Smith C. (2005). Genomics: SNPs and human disease. Nature.

[55] Singh M., Singh P., Juneja P.K., Singh S., Kaur T. (2011). SNP–SNP interactions within APOE gene influence plasma lipids in postmenopausal osteoporosis. Rheumatol. Int..

[56] Sapkota Y., Mackey J.R., Lai R., Franco-Villalobos C., Lupichuk S., Robson P.J., Kopciuk K., Cass C.E., Yasui Y., Damaraju S. (2013). Assessing SNP-SNP interactions among DNA repair, modification and metabolism related pathway genes in breast cancer susceptibility. PLoS ONE.

[57] Onay V.Ü., Briollais L., Knight J.A., Shi E., Wang Y., Wells S., Li H., Rajendram I., Andrulis I.L., Ozcelik H. (2006). SNP-SNP interactions in breast cancer susceptibility. BMC Cancer.

[58] Moszyńska A., Gebert M., Collawn J.F., Bartoszewski R. (2017). SNPs in microRNA target sites and their potential role in human disease. Open Biol..

[59] Soares-Silva M., Diniz F.F., Gomes G.N., Bahia D. (2016). The mitogen-activated protein kinase (MAPK) pathway: Role in immune evasion by trypanosomatids. Front. Microbiol..

[60] Parihar S., Ozturk M., Marakalala M., Loots D., Hurdayal R., Maasdorp D.B., Van Reenen M., Zak D., Darboe F., Penn-Nicholson A. (2018). Protein kinase C-delta (PKC*δ*), a marker of inflammation and tuberculosis disease progression in humans, is important for optimal macrophage killing effector functions and survival in mice. Mucosal Immunol..

[61] Berg L.J., Finkelstein L.D., Lucas J.A., Schwartzberg P.L. (2005). Tec family kinases in T lymphocyte development and function. Annu. Rev. Immunol..

[62] Felices M., Falk M., Kosaka Y., Berg L.J. (2007). Tec kinases in T cell and mast cell signaling. Adv. Immunol..

[63] Readinger J.A., Mueller K.L., Venegas A.M., Horai R., Schwartzberg P.L. (2009). Tec kinases regulate T-lymphocyte development and function: New insights into the roles of Itk and Rlk/Txk. Immunol. Rev..

[64] Fan K., Jia Y., Wang S., Li H., Wu D., Wang G., Chen J.L. (2012). Role of Itk signalling in the interaction between influenza A virus and T-cells. J. Gen. Virol..

[65] Clements J.L., Ross-Barta S.E., Tygrett L.T., Waldschmidt T.J., Koretzky G.A. (1998). SLP-76 expression is restricted to hemopoietic cells of monocyte, granulocyte, and T lymphocyte lineage and is regulated during T cell maturation and activation. J. Immunol..

[66] Koretzky G.A., Abtahian F., Silverman M.A. (2006). SLP76 and SLP65: Complex regulation of signalling in lymphocytes and beyond. Nat. Rev. Immunol..

[67] Xu H., Littman D.R. (1993). A kinase-independent function of Lck in potentiating antigen-specific T cell activation. Cell.

[68] Lattanzio R., Iezzi M., Sala G., Tinari N., Falasca M., Alberti S., Buglioni S., Mottolese M., Perracchio L., Natali P.G. (2019). PLC-gamma-1 phosphorylation status is prognostic of metastatic risk in patients with early-stage Luminal-A and-B breast cancer subtypes. BMC Cancer.

[69] Yang Y.R., Choi J.H., Chang J.S., Kwon H.M., Jang H.J., Ryu S.H., Suh P.G. (2012). Diverse Cellular and Physiological Roles of Phospholipase C-γ1.

[70] Herman J.A., Miller M.P., Biggins S. (2020). chTOG is a conserved mitotic error correction factor. Elife.

[71] Jang C.W., Shibata Y., Starmer J., Yee D., Magnuson T. (2015). Histone H3. 3 maintains genome integrity during mammalian development. Genes Dev..

[72] Weaver D.C., Shpakovski G.V., Caputo E., Levin H.L., Bocke J. (1993). Sequence analysis of closely related retrotransposon families from fission yeast. Gene.

[73] Mellor P., Furber L.A., Nyarko J.N., Anderson D.H. (2012). Multiple roles for the p85*α* isoform in the regulation and function of PI3K signalling and receptor trafficking. Biochem. J..

[74] Chagpar R.B., Links P.H., Pastor M.C., Furber L.A., Hawrysh A.D., Chamberlain M.D., Anderson D.H. (2010). Direct positive regulation of PTEN by the p85 subunit of phosphatidylinositol 3-kinase. Proc. Natl. Acad. Sci. USA.

[75] Morchikh M., Cribier A., Raffel R., Amraoui S., Cau J., Severac D., Dubois E., Schwartz O., Bennasser Y., Benkirane M. (2017). HEXIM1 and NEAT1 long non-coding RNA form a multi-subunit complex that regulates DNA-mediated innate immune response. Mol. Cell.

[76] Mascareno E., Gupta R., Martello L.A., Dhar-Mascareno M., Salciccioli L., Beckles D., Walsh M.G., Machado F.S., Tanowitz H.B., Haseeb M. (2018). Rapidly progressive course of Trypanosoma cruzi infection in mice heterozygous for hexamethylene bis-acetamide inducible 1 (Hexim1) gene. Microbes Infect..

[77] Vousden K.H., Prives C. (2009). Blinded by the light: The growing complexity of p53. Cell.

[78] Efeyan A., Serrano M. (2007). p53: Guardian of the genome and policeman of the oncogenes. Cell Cycle.

[79] Komarova E.A., Krivokrysenko V., Wang K., Neznanov N., Chernov M.V., Komarov P.G., Brennan M.L., Golovkina T.V., Rokhlin O., Kuprash D.V. (2005). p53 is a suppressor of inflammatory response in mice. FASEB J..

[80] Madenspacher J.H., Azzam K.M., Gowdy K.M., Malcolm K.C., Nick J.A., Dixon D., Aloor J.J., Draper D.W., Guardiola J.J., Shatz M. (2013). p53 Integrates host defense and cell fate during bacterial pneumonia. J. Exp. Med..

[81] Chung J.H. (2018). The role of DNA-PK in aging and energy metabolism. FEBS J..

[82] Ferguson B.J., Mansur D.S., Peters N.E., Ren H., Smith G.L. (2012). DNA-PK is a DNA sensor for IRF-3-dependent innate immunity. Elife.

[83] Black J.A., Crouch K., Lemgruber L., Lapsley C., Dickens N., Tosi L.R., Mottram J.C., McCulloch R. (2020). *Trypanosoma brucei* ATR links DNA damage signaling during antigenic variation with regulation of RNA polymerase I-transcribed surface antigens. Cell Rep..

[84] Baker N., Catta-Preta C.M., Neish R., Sadlova J., Powell B., Alves-Ferreira E.V., Geoghegan V., Carnielli J.B., Newling K., Hughes C. (2021). Systematic functional analysis of Leishmania protein kinases identifies regulators of differentiation or survival. Nat. Commun..

[85] Xu J., Cai R., Lu L., Duan C., Tao X., Chen D., Liu Y., Wang X., Cao M., Chen Y. (2014). Genetic regulatory network analysis reveals that low density lipoprotein receptor-related protein 11 is involved in stress responses in mice. Psychiatry Res..

[86] Seguel M., Perez-Venegas D., Gutierrez J., Crocker D.E., DeRango E.J. (2019). Parasitism elicits a stress response that allocates resources for immune function in South American fur seals (*Arctocephalus australis*). Physiol. Biochem. Zool..

[87] Fleming A. (1922). On a remarkable bacteriolytic element found in tissues and secretions. Proc. R. Soc. London Ser. Contain. Pap. Biol. Character.

[88] Ng C.J., Wadleigh D.J., Gangopadhyay A., Hama S., Grijalva V.R., Navab M., Fogelman A.M., Reddy S.T. (2001). Paraoxonase-2 is a ubiquitously expressed protein with antioxidant properties and is capable of preventing cell-mediated oxidative modification of low density lipoprotein. J. Biol. Chem..

[89] Bourquard N., Ng C.J., Reddy S.T. (2011). Impaired hepatic insulin signalling in PON_2_-deficient mice: A novel role for the PON_2_/apoE axis on the macrophage inflammatory response. Biochem. J..

[90] Devarajan A., Bourquard N., Grijalva V.R., Gao F., Ganapathy E., Verma J., Reddy S.T. (2013). Role of PON_2_ in innate immune response in an acute infection model. Mol. Genet. Metab..

[91] Sprecher C.A., Grant F.J., Baumgartner J.W., Presnell S.R., Schrader S.K., Yamagiwa T., Whitmore T.E., O’Hara P.J., Foster D.F. (1998). Cloning and characterization of a novel class I cytokine receptor. Biochem. Biophys. Res. Commun..

[92] Pflanz S., Hibbert L., Mattson J., Rosales R., Vaisberg E., Bazan J.F., Phillips J.H., McClanahan T.K., de Waal Malefyt R., Kastelein R.A. (2004). WSX-1 and glycoprotein 130 constitute a signal-transducing receptor for IL-27. J. Immunol..

[93] Villarino A., Hibbert L., Lieberman L., Wilson E., Mak T., Yoshida H., Kastelein R.A., Saris C., Hunter C.A. (2003). The IL-27R (WSX-1) is required to suppress T cell hyperactivity during infection. Immunity.

[94] Reczek D., Schwake M., Schröder J., Hughes H., Blanz J., Jin X., Brondyk W., Van Patten S., Edmunds T., Saftig P. (2007). LIMP-2 is a receptor for lysosomal mannose-6-phosphate-independent targeting of *β*-glucocerebrosidase. Cell.

[95] Neculai D., Schwake M., Ravichandran M., Zunke F., Collins R.F., Peters J., Neculai M., Plumb J., Loppnau P., Pizarro J.C. (2013). Structure of LIMP-2 provides functional insights with implications for SR-BI and CD36. Nature.

[96] Heybrock S., Kanerva K., Meng Y., Ing C., Liang A., Xiong Z.J., Weng X., Kim Y.A., Collins R., Trimble W. (2019). Lysosomal integral membrane protein-2 (LIMP-2/SCARB2) is involved in lysosomal cholesterol export. Nat. Commun..

[97] Mori H., Sakakibara S.i., Imai T., Nakamura Y., Iijima T., Suzuki A., Yuasa Y., Takeda M., Okano H. (2001). Expression of mouse igf2 mRNA-binding protein 3 and its implications for the developing central nervous system. J. Neurosci. Res..

[98] Natkunam Y., Vainer G., Chen J., Zhao S., Marinelli R.J., Hammer A.S., Hamilton-Dutoit S., Pikarsky E., Amir G., Levy R. (2007). Expression of the RNA-binding protein VICKZ in normal hematopoietic tissues and neoplasms. Haematologica.

[99] Patel S.B., Bellini M. (2008). The assembly of a spliceosomal small nuclear ribonucleoprotein particle. Nucleic Acids Res..

[100] Luo H.R., Moreau G.A., Levin N., Moore M.J. (1999). The human Prp8 protein is a component of both U2-and U12-dependent spliceosomes. RNA.

[101] Lee S.Y., Park E.J., Kim S., Lee C.W. (2018). Ssu72 Phosphatase Is Involved in Immunometabolism. J. Immunol..

[102] Silva J.S., Twardzik D.R., Reed S.G. (1991). Regulation of Trypanosoma cruzi infections in vitro and in vivo by transforming growth factor beta (TGF-beta). J. Exp. Med..

[103] Wang L., Li Q., Ni S., Huang Y., Wei J., Liu J., Yu Y., Wang S., Qin Q. (2019). The roles of grouper clathrin light chains in regulating the infection of a novel marine DNA virus, Singapore grouper iridovirus. Sci. Rep..

[104] Ni H., Wang X.S., Diener K., Yao Z. (1998). MAPKAPK5, a novel mitogen-activated protein kinase (MAPK)-activated protein kinase, is a substrate of the extracellular-regulated kinase (ERK) and p38 kinase. Biochem. Biophys. Res. Commun..

[105] Kress T.R., Cannell I.G., Brenkman A.B., Samans B., Gaestel M., Roepman P., Burgering B.M., Bushell M., Rosenwald A., Eilers M. (2011). The MK5/PRAK kinase and Myc form a negative feedback loop that is disrupted during colorectal tumorigenesis. Mol. Cell.

[106] Casacuberta-Serra S., Soucek L. (2018). Myc and Ras, the Bonnie and Clyde of immune evasion. Transl. Cancer Res..

[107] Okada M. (2012). Regulation of the SRC family kinases by Csk. Int. J. Biol. Sci..

[108] Niki M., Di Cristofano A., Zhao M., Honda H., Hirai H., Van Aelst L., Cordon-Cardo C., Pandolfi P.P. (2004). Role of Dok-1 and Dok-2 in leukemia suppression. J. Exp. Med..

[109] Kim S.Y., Kim S., Bae D.J., Park S.Y., Lee G.Y., Park G.M., Kim I.S. (2017). Coordinated balance of Rac1 and RhoA plays key roles in determining phagocytic appetite. PLoS ONE.

[110] Wang L., Liu Y., Beier U.H., Han R., Bhatti T.R., Akimova T., Hancock W.W. (2013). Foxp3+ T-regulatory cells require DNA methyltransferase 1 expression to prevent development of lethal autoimmunity. Blood J. Am. Soc. Hematol..

