# Supplementary material for: Deciphering the Molecular Mechanism Underlying African Animal Trypanosomiasis by Means of the 1000 Bull Genomes Project Genomic Dataset
Source: Biology (Basel). 2022 May 13;11(5):742. doi: 10.3390/biology11050742 (PMC9138820; doi:10.3390/biology11050742)
Supplement: Supplementary file 1 [file biology-11-00742-s001.zip › SupplementaryFileS4.pdf]

Supplementary Information

Table showing the effectors and their corresponding monotonically expressed genes which harbour regulatory SNP in their promoter regions

Cattle breed	Tissue	Effectors	Key regulatory MEGs harbouring rSNPs
Boran	Liver	Itk:Lck:PLCgamma1:SLP-76	*MAPKAPK5*
Boran	Liver	PKCdelta	*CSK, DOK2, RAC1, DNMT1*
Boran	Liver	SRF	*CSK, DOK2, RAC1, DNMT1*
N'Dama	Liver	CHTOG:h3f3a	*CSK, DOK2, RAC1, DNMT1*
N'Dama	Liver	p85alpha	*CSK, DOK2, RAC1, DNMT1*
N'Dama	Liver	TFII-I	*CSK, DOK2, RAC1, DNMT1*

Gene expression profiles of monotonically expressed genes harbouring regulatory SNP in their promoter regions

Visualisation of the expression values of monotonically expressed genes. In the title of the figures below, gene ID represents the Ensembl ID of the genes and gene symbols refer to the gene names. Red lines in the figure correspond to the gene expression values of the cattle breed Boran and the green lines correspond to N'Dama. In the following plots, y-axis represents the gene expression values and x-axis represents the time points day 0, day 12, day 15, day 18, day 21, day 26, day 32 and day 35 after trypanosome infection.

FIGURE S1

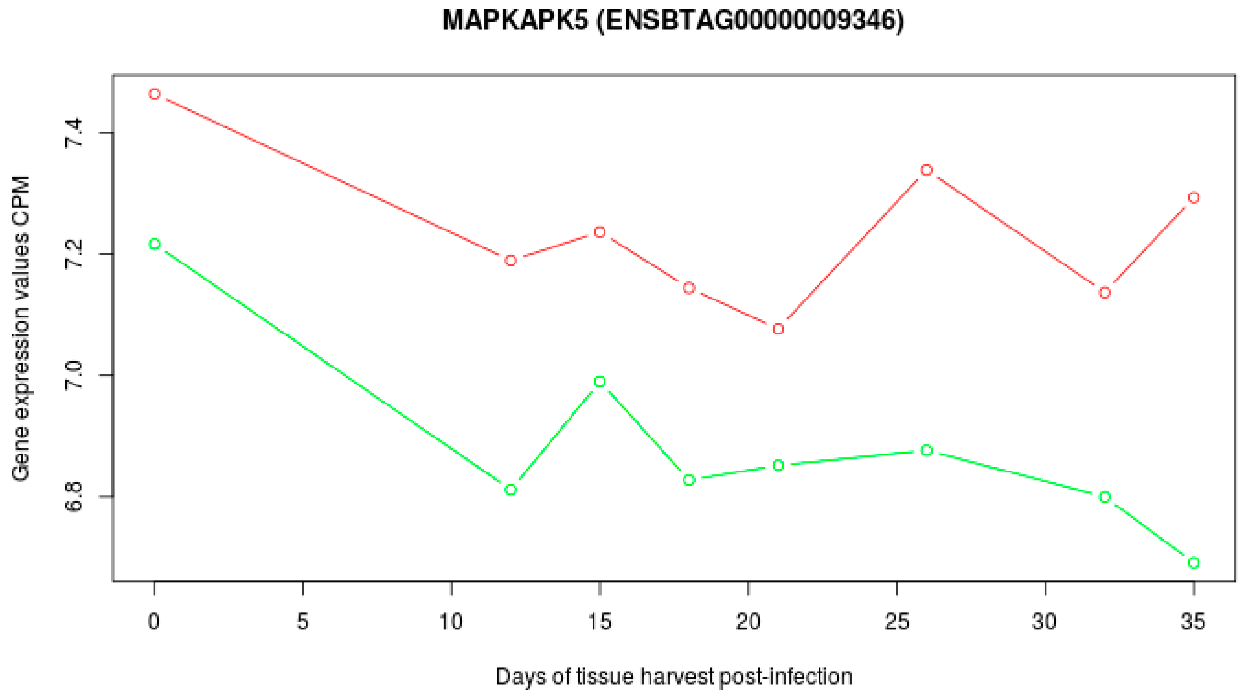

FIGURE S2

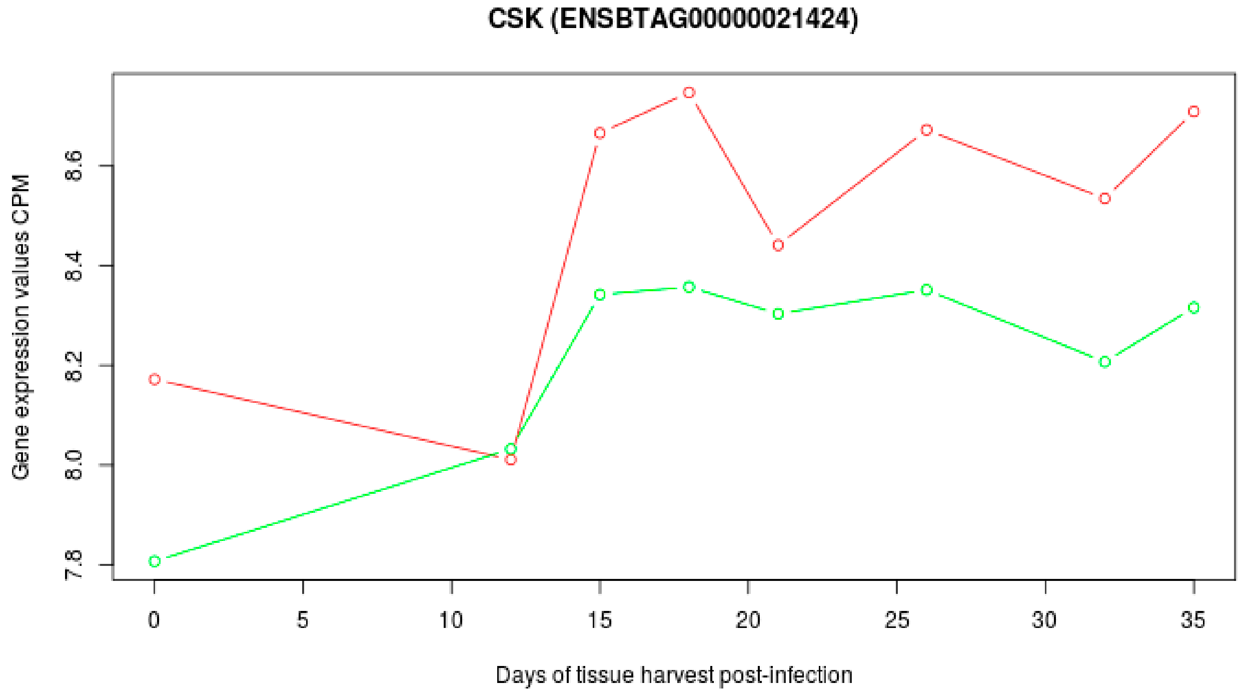

FIGURE S3

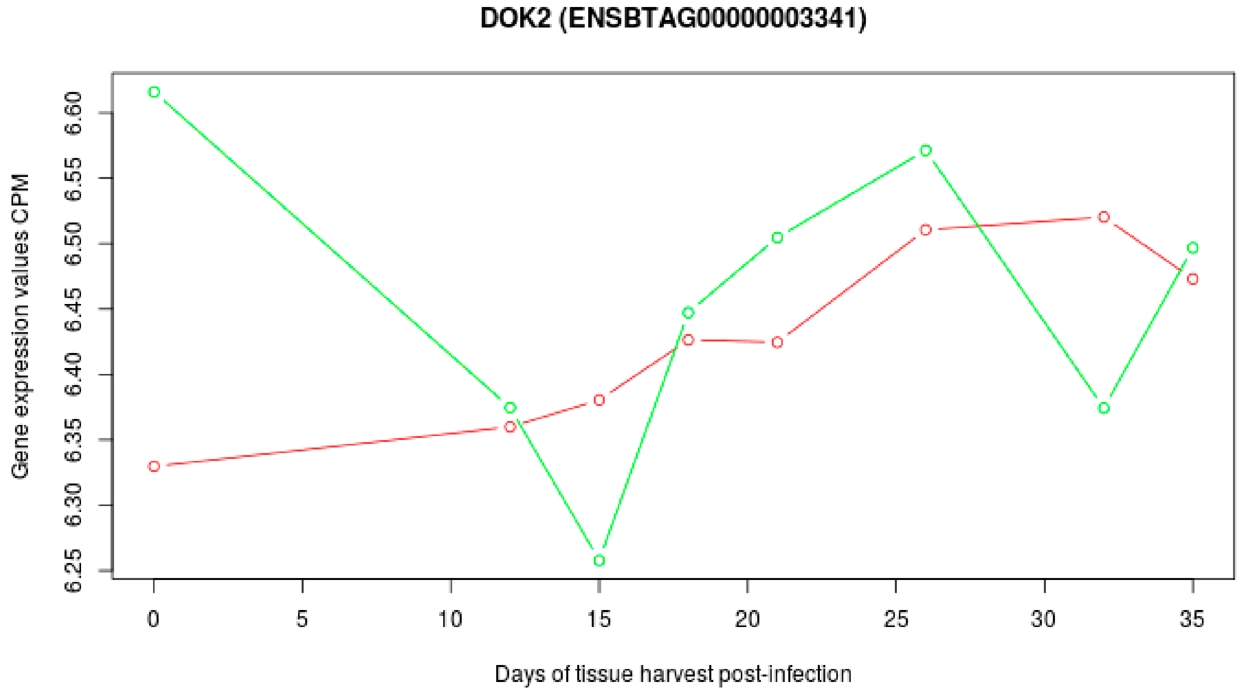

FIGURE S4

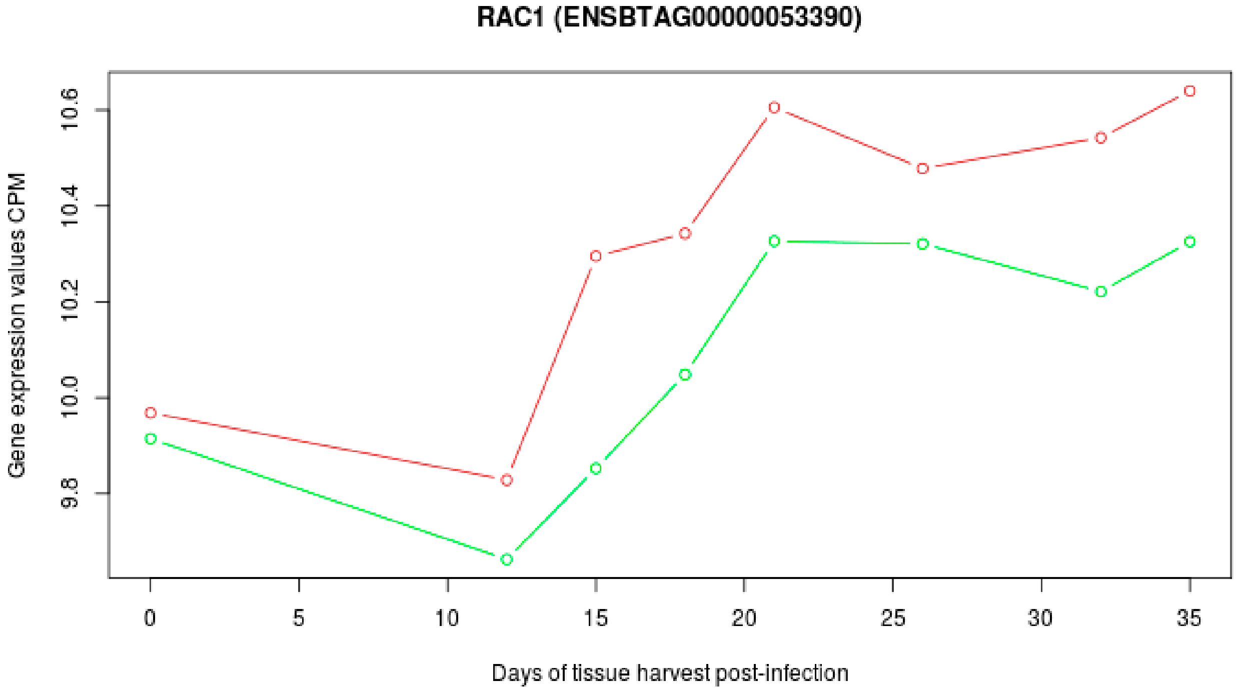

FIGURE S5

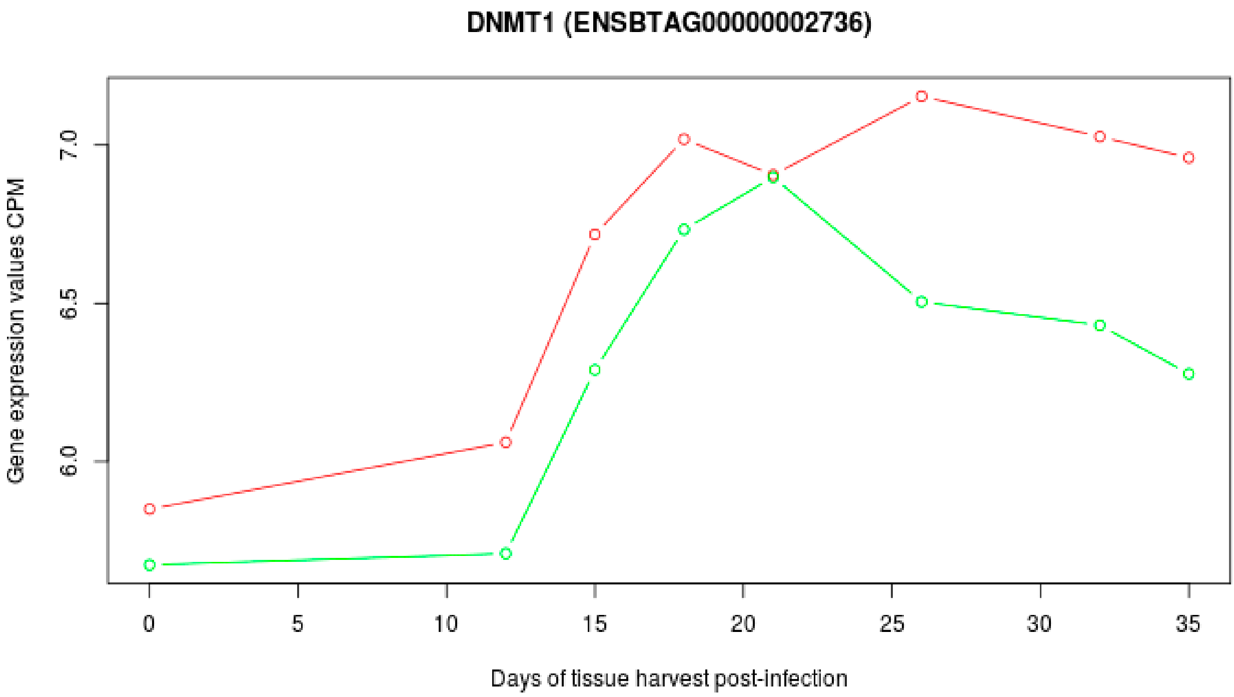

DNMT1 (ENSBTAG00000002736)
Gene expression values CPM
6.0
6.5
7.0
0
5
10
15
20
25
30
35
Days of tissue harvest post-infection
