# Supplementary material for: Deciphering the Molecular Mechanism Underlying African Animal Trypanosomiasis by Means of the 1000 Bull Genomes Project Genomic Dataset
Source: Biology (Basel). 2022 May 13;11(5):742. doi: 10.3390/biology11050742 (PMC9138820; doi:10.3390/biology11050742)
Supplement: Supplementary file 1 [file biology-11-00742-s001.zip › SupplementaryFileS5.pdf]

Supplementary Information

QR codes consisting of gene expression profiles for monotonically expressed genes harboring regulatory SNP in their promoter regions

Visualization of the expression values of monotonically expressed genes. In the title of the figures below, gene ID represents the Ensembl ID of the genes and gene symbols refer to the gene names. Red lines in the figure correspond to the gene expression values of the cattle breed Boran and the green lines correspond to N'Dama. In the following plots of liver tissue, y-axis represents the gene expression values and x-axis represents the time points day 0, day 12, day 15, day 18, day 21, day 26, day 32 and day 35 after trypanosome infection. On the other hand, in the plots of spleen and lymph node tissue, y-axis represents the gene expression values and x-axis represents the time points day 0, day 21, and day 35 after trypanosome infection.

1. Liver tissue of Boran

   Gene expression profiles of monotonically expressed genes harboring regulatory SNP leading to “Gain of TFBS” in their promoter regions

   https://drive.google.com/file/d/1Z79W6EJxeLGpJQ_IiuAEZw2PnRFMl3ui/view?usp=sharing

2. Liver tissue of Boran

   Gene expression profiles of monotonically expressed genes harboring regulatory SNP leading to “Loss of TFBS” in their promoter regions

   https://drive.google.com/file/d/1SqXZyjvP1iCPEDk9HyNBVWIVhBS2ZDh8/view?usp=sharing

3. Liver tissue of N’Dama

   Gene expression profiles of monotonically expressed genes harboring regulatory SNP leading to “Gain of TFBS” in their promoter regions

   https://drive.google.com/file/d/16NIA_vT6kLmFVEpE0hPyQ9OKvHnn1shq/view?usp=sharing

4. Liver tissue of N’Dama

   Gene expression profiles of monotonically expressed genes harboring regulatory SNP leading to “Loss of TFBS” in their promoter regions

   https://drive.google.com/file/d/1YJUtVFj_vmWaWY3RMa-mNfHLQ1D4xkw6/view?usp=sharing

5. Spleen tissue of Boran

   Gene expression profiles of monotonically expressed genes harboring regulatory SNP leading to “Gain of TFBS” in their promoter regions

   https://drive.google.com/file/d/1MpeIBVQNyk02kDCH_IcC9tKE-ouF0MYU/view?usp=sharing

6. Spleen tissue of Boran

   Gene expression profiles of monotonically expressed genes harboring regulatory SNP leading to “Loss of TFBS” in their promoter regions

   https://drive.google.com/file/d/1UY3B1vZz9O6X2UaXOlFyQ5e5lhwiy5hV/view?usp=sharing

7. Spleen tissue of N'Dama

   Gene expression profiles of monotonically expressed genes harboring regulatory SNP leading to "Loss of TFBS" in their promoter regions

   https://drive.google.com/file/d/1oUWIzKrsmEfzbb9i2p6ioxyMTuwqWC-9/view?usp=sharing

8. Lymph node tissue of Boran

   Gene expression profiles of monotonically expressed genes harboring regulatory SNP leading to "Gain of TFBS" in their promoter regions

   https://drive.google.com/file/d/1FdFolx3NrM02rv0d28w9qpOpyFyay7oJ/view?usp=sharing

9. Lymph node tissue of Boran

   Gene expression profiles of monotonically expressed genes harboring regulatory SNP leading to “Loss of TFBS” in their promoter regions

   https://drive.google.com/file/d/1tnTdq9Cjnlxkyk_3BVACVMSZMuufAILU/view?usp=sharing

10. Lymph node tissue of N’Dama

   Gene expression profiles of monotonically expressed genes harboring regulatory SNP leading to “Loss of TFBS” in their promoter regions

   https://drive.google.com/file/d/1vQda2E--APNijJJC2N1yjwQxbF8qXuUb/view?usp=sharing
